# MiR-455-3p inhibits gastric cancer progression by repressing Wnt/β-catenin signaling through binding to ARMC8

**DOI:** 10.1186/s12920-023-01583-y

**Published:** 2023-07-03

**Authors:** Ting Zhan, Mengge Chen, Weijie Liu, Zheng Han, Qingxi Zhu, Meng Liu, Jie Tan, Jiaxi Liu, Xiaoli Chen, Xia Tian, Xiaodong Huang

**Affiliations:** 1 Department of Gastroenterology, WuHan Third Hospital ( Tongren hospital of WuHan University), Wuhan, 430060 China; 2 Department of Gastroenterology, Zhongnan Hospital of Wuhan University, Wuhan, 430060 China

**Keywords:** miR-455-3p, ARMC8, Progression, Wnt/βcatenin, Gastric cancer

## Abstract

**Background:**

Globally, gastric cancer (GC) is one of the world’s most widespread malignancies, with persistent high mortality and morbidity rates. Increasing evidence now suggests that microRNAs (miRNAs) participate in many biological processes, with miR-455-3p having key roles in the progression of diverse cancers. Nevertheless, miR-455-3p function and expression in GC remain unclear.

**Methods:**

We explored miR-455-3p expression in GC using quantitative polymerase chain reaction (qPCR). To further examine the effect of miR-455-3p in GC, after transfecting miR-455-3p mimics or inhibitors into GC cells, 5-ethynyl-2’-deoxyuridine (EdU) incorporation and colony formation assays were performed to examine cell proliferation. Flow cytometry was used to detect apoptosis, and expression levels of Bax, Bcl-2, Snail, N-cadherin, E-cadherin, and Caspase-3 were assessed by western blotting (WB). Using online databases and luciferase assays, we identified armadillo repeat-containing protein 8 (ARMC8) as a promising target of miR-455-3p. A mouse tumor model was established to investigate actions of miR-455-3p in vivo. Expression levels of C-myc, cyclinD1, and β-catenin were examined using WB and immunofluorescence.

**Results:**

MiR-455-3p expression was attenuated in GC tissue and cell lines. MiR-455-3p overexpression inhibited GC cell proliferation, epithelial-mesenchymal transition (EMT), as well as facilitated apoptosis, while suppression of miR-455-3p had the opposite effects. From luciferase assays, we confirmed that ARMC8 was a novel and direct downstream target gene of miR-455-3p, and that the tumor suppressive role of miR-455-3p was in part reversed due to ARMC8 overexpression. Moreover, miR-455-3p inhibited GC growth in vivo via ARMC8. We also observed that miR-455-3p repressed canonical Wnt pathway activation by binding to ARMC8.

**Conclusions:**

MiR-455-3p exerted tumor inhibitory effects in GC by targeting ARMC8. Therefore, intervening in the miR-455-3p/ARMC8/Wnt/βcatenin axis could be a promising novel treatment strategy for GC.

**Supplementary Information:**

The online version contains supplementary material available at 10.1186/s12920-023-01583-y.

## Introduction

Globally, gastric cancer (GC) is a leading cause of cancer death [1], but despite declining morbidity and mortality rates in recent decades, high metastasis rates generate poor prognosis outcomes for GC [2]. In particular, for advanced GC, successful early diagnosis and surgical resection rates are very low [3]. Therefore, characterizing genes closely related to GC pathophysiology is crucial, especially for early clinical diagnosis and targeted therapy outcomes.

MiRNAs are small non-coding 19–24 nucleotide RNAs, which typically bind to complementary sequences in the 3′-untranslated regions (UTRs) of mRNAs to modulate gene expression [4]. As post-transcriptional regulatory factors, miRNAs modulate diverse physiological processes, including apoptosis, proliferation, EMT, autophagy, and other regulatory processes [5]. MiRNAs are aberrantly expressed in GC tissue and implicated in GC tumorigenesis [6]. MiR-455-3p is associated with the occurrence, development, and prognosis of esophageal [7], colorectal [8], breast [9], pancreatic [10], and hepatocellular cancers [11]. However, no studies have yet characterized miR-455-3p functions in GC.

The ARMC family of proteins contains tandem repeat sequences of approximately 42 amino acids in length [12]. Several reports have shown that ARMC8 forms a critical portion of the human C-terminal leptospirosis-1 type homology complex [13], which is linked to several physiological processes, features in Wnt, phosphoinositide 3-kinase (PI3K), NF-kappaB (NF-κB), and transforming growth factor β (TGF-β) pathways, and is linked to cell adhesion, proliferation, and migration [14]. ARMC8 is overexpressed in many cancers, and is connected to negative prognoses, lymph node metastasis, and tumor-node-metastasis stages [15–18]. However, the mechanisms and actions underlying ARMC8 in GC requires investigation.

In this study, we analyzed miR-455-3p expression data from The Cancer Genome Atlas (TCGA). We identified the expression of miR-455-3p was attenuated in GC tissue and cell lines. Inhibition of miR-455-3p expression enhanced EMT and GC cell proliferation, but suppressed apoptosis. Overexpression of miR-455-3p repressed GC progression in vivo. Additionally, online databases were used to predict target genes of miR-455-3p. We identified ARMC8 and showed that miR-455-3p acted as a direct post-transcriptional modulator of ARMC8 and that miR-455-3p had anti-tumor roles by regulating the ARMC8/Wnt/β-catenin axis in GC. Therefore, we provide novel insights into the molecular mechanisms underpinning GC.

## Materials and methods

### Data and clinical information mining and processing

The mRNA-sequencing and miRNA-sequencing data of stomach adenocarcinoma (STAD) were retrieved from The Cancer Genome Atlas (TCGA) database portal (https://portal.gdc.cancer.gov/), The database contains 375 gastric cancer tissues, of which 32 had paired adjacent tissues. Thirty-two paired GC tissue and adjacent tissue data were used to analyze the differential expression of miR-455-3p and ARMC8. The ARMC8 mRNA sequencing data from 375 GC tissues were used for gene set enrichment analysis (GSEA).

### Target gene prediction

miRDB, mirDIP, miRSystem and TargetScan databases were used to identify potential target gene of miR-455-3p. Afterwards, we intersected the results from the four databases via Venn plotting web-site (http://bioinformatics.psb.ugent.be/webtools/Venn/). Because ARMC8 has high target scores in the miRDB database, ARMC8 was selected from the 49 target genes intersected in the four databases mentioned above.

### Patient tissue specimens

In this study, 13 paired human GC and corresponding noncancerous samples were collected from patients who had received surgical treatment in Wuhan Third Hospital. All enrolled samples did not receive any neoadjuvant chemotherapy before surgical treatment. These specimens were stored in liquid nitrogen at -80 °C as soon as possible until RNA extraction. All methods were been performed in accordance with the Declaration of Helsinki and were conducted under the guidelines provided by the Wuhan Third Hospital Ethics Committee (Ethics number:2021-013). Informed consent was obtained from all subjects and/or their legal guardian(s).

### Cell lines and cell culture

Human GC cell lines HGC-27 and AGS were obtained from the Cell Bank of Type Culture Collection of the Chinese Academy of Sciences (Shanghai, China). It was first placed in medium containing 10% fetal bovine serum (NQBB, USA), 100 mg/mL streptomycin and 100 IU/mL penicillin (Thermo Fisher Scientific, USA) in RPMI1640 medium (Gibco, USA) and then cultured in a humidified incubator at 37 °C with 5% CO2. All methods were been performed in accordance with the *Declaration of Helsinki.*

### Cell transfection

Hsa-miR-455-3p mimic (sense: 5’-GCAGUCCAUGGGCAUAUACAC-3’; antisense: 5’-GUAUAUGCCCAUGGACUGCUU-3’), hsa-miR-455-3p inhibitor (sense: 5’-GUGUAUAUGCCCAUGGACUGC-3’), hsa-miR mimic NC (sense: 5’-UUCUCC GAACGUGUCACGUTT-3’; anti-sense:5’-ACGUGACACGUUCGGAGAATT-3’), and hsa-miR inhibitor NC (5’-CAGUACUUUUGUGUAGUACAA-3’) were synthesized by Genepharma (Shanghai, China). 455-3p mimics (40 nM), miR-455-3p inhibitors (80 nM) or their NC were transfected into HGC-27 and AGS cells using Lipofectamine 2000 (Invitrogen, USA) following the manufacturer’s instructions. The ARMC8 lentiviral expression vector (lenti-ARMC8) was provided by GeneChem (Shanghai, China), transfected based on the manufacturer’s guidelines.

### Total RNA extraction and qPCR analysis

Total RNA was extracted from the above-mentioned GC tissues and cell lines using TRIzol reagent (Invitrogen, USA), followed by cDNA synthesis based on the manufacturer’s instructions (TOYOBO, Japan). To investigate mRNA expression levels of ARMC8, reverse transcription results were performed using UltraSYBR Mixture (ComWin Biotech, China) on an ABI StepOne Plus qPCR system (Applied Biosystems, USA). The endogenous controls for the assays were GAPDH mRNA. To estimate the expression of miR-455-3p, we conducted inverse transcription to synthesize cDNA from RNA using the PrimeScript RT Reagent kit (Takara Bio, Japan). QRT-PCR analysis was carried out with ABI PRISM 7500 Real-Time PCR System (Applied Biosystems). Endogenous U6 (Ribobio, China), a small nuclear RNA (snRNA), was used for normalization. The cycling protocol was as follows: hot-start at 95°C for 5 min; followed by 40 cycles of 95°C denaturation and 60°C annealing. The 2^−ΔΔ^CT method was used to calculate changes in mRNA and miRNA expression levels. The primers for amplification were as follows: ARMC8(F, 5’- TCGATGACCCTGGTAAATG − 3’, and R, 5’- CTGTCCGAATTGCTCCA − 3’); GAPDH (F: 5’ -TCTGACTTCAACAGCGACAC-3’; R: 5’-CAAATTCGTTGTCATACCAG-3’); miR-455-3p (RT, 5’- CTCAACTGGTGTCGTGGAGTCGGCAATTCAGTTGAGGTGTAT − 3’; F, 5’- ACACTCCAGCTGGGGCAGTCCATGGGCAT-3’); U6(RT, 5’-AACGCTTCACGAATTTGCGT-3’; F, 5’- CTCGCTTCGGCAGCACA − 3’).

### Western blot analysis

GC cells were placed in radioimmunoprecipitation assay (RIPA) buffer containing protease inhibitor (Beyotime, Shanghai, China) to allow protein release, followed by protein concentration measurement using Pierce Rapid Gold BCA Protein Assay Kit (Elabscience, USA). Proteins were separated via 10% SDS-PAGE, followed by transfer to NC membranes and then incubated with primary antibodies against ARMC8.(1:1000, CST, USA), Bcl-2 (1:1000, abcam, USA), caspase-3(1:1000, CST), Bax (1:1000, CST), snail (1:1000, abcam), E-cadherin (1:1000, abcam), N-cadherin (1:1000, abcam), b-catenin (1:1000; Abcam), c-myc (1:1000; Abcam), cyclin D1 (1:1000; Abcam), and β-actin (1:1000, Santa Cruz, Delaware, CA) overnight at 4 °C. It was subsequently incubated with secondary antibodies (LI-COR Biosciences, USA) and protein levels were assessed by an enhanced chemiluminescence (ECL) Western Blot analytical detection system (Amersham, USA).

### EdU incorporation assays

The BeyoClick™ EdU Cell Proliferation Assay Kit (Beyotime, Shanghai, China) was performed to assess the proliferation of transfected GC cells based on the manufacturer’s instructions. For quantitative measurement of cell proliferation, EdU incorporation was assessed via DAB staining following the manufacturer’s protocol.

### Colony formation assay

Colony Formation Assay was performed as described [10].

### Analysis of cell apoptosis

After transfection and incubation for 48 h, gastric cancer cells were stained with the Annexin V-PE assay kit (BD Biosciences, USA) based on the manufacturer’s instructions. All specimens were performed using the FACS Caliber II sorter and the Cell Quest FACS system (BD Biosciences, USA) for analysis.

### Luciferase activity assay

The wild-type (wt) or mutant (mt) 3’-UTR sequence of ARMC8 was inserted into the downstream of the reporter gene between the XbaI and Not I (New England Biolabs, NEB, Ipswich, MA, England) loci of the pRL-TK vector (Promega, Madison, WI, USA), and successful cloning was identified by Sanger DNA sequencing. After seeding AGS and HGC-27 cells in 24-well plates, it was co-transfected with miR-455-3p mimic or mimic NC and the psiCHECK2-UTR vector (Promega, USA) via Lipofectamine 2000 (Invitrogen, USA). Finally, a dual-Luciferase Reporter Assay System (Promega, USA) was performed to measure luciferase activity.

### Tumor formation in Xenograft Model

The 5-week-old male BALB/c nude mice (18–20 g) were purchased from Beijing Vital River Laboratory Animal Technology (Beijing, China) and randomly divided into two group of five each (one group for agomir NC and another group for miR-455-3p agomir). A total of 5*10^6^ HGC-27 cells were resuspended in 100 mL of PBS-containing 25% Matrigel were subcutaneously injected into the flank of the nude mice. Once the tumor volume in nude mice grew to about 100mm^3^, miR-455-3p agomir and agomir NC (GenePharma, Shanghai, China) were injected into multiple different sites of the tumor thrice every 5 days for a total of 3 injections. Tumor size was measured every 5 days. All nude mice were executed and dissected after 30 days to obtain tumor tissue for subsequent experimental analysis. The tumor size was observed by measuring the length (L) and width (W) with calipers and calculated as follows: tumor size = L*W^2^/2. This work meets requirements of the animal experiment ethics committee of Wuhan Third Hospital. All methods are reported in accordance with ARRIVE guidelines (https://arriveguidelines.org) for the reporting of animal experiments.

### Histological analysis

These tumor tissues were first received for pathological sectioning. The primary antibodies performed for immunohistochemistry analysis were ARMC8 antibody (1:100, CST, USA) and Ki67 antibody (1:100, CST, USA).

### Immunofluorescence

Immunofluorescence experimental steps refer to [19].

### Statistical analysis

Statistical analysis was performed with SPSS 22.0 soft-ware and illustration data were presented by GraphPad Prism 8.0.2. Enumeration data are expressed as ratios or percentages, and measurement data are expressed as mean ± standard deviation. Differences between GC tissues and corresponding noncancerous tissues were compared by paired t test, whereas other sample comparisons were made using independent sample t-tests. *P* < 0.05 was considered was consider as a statistical reference value.

## Results

### MiR-455-3p expression is altered in GC

**Fig. 1:**
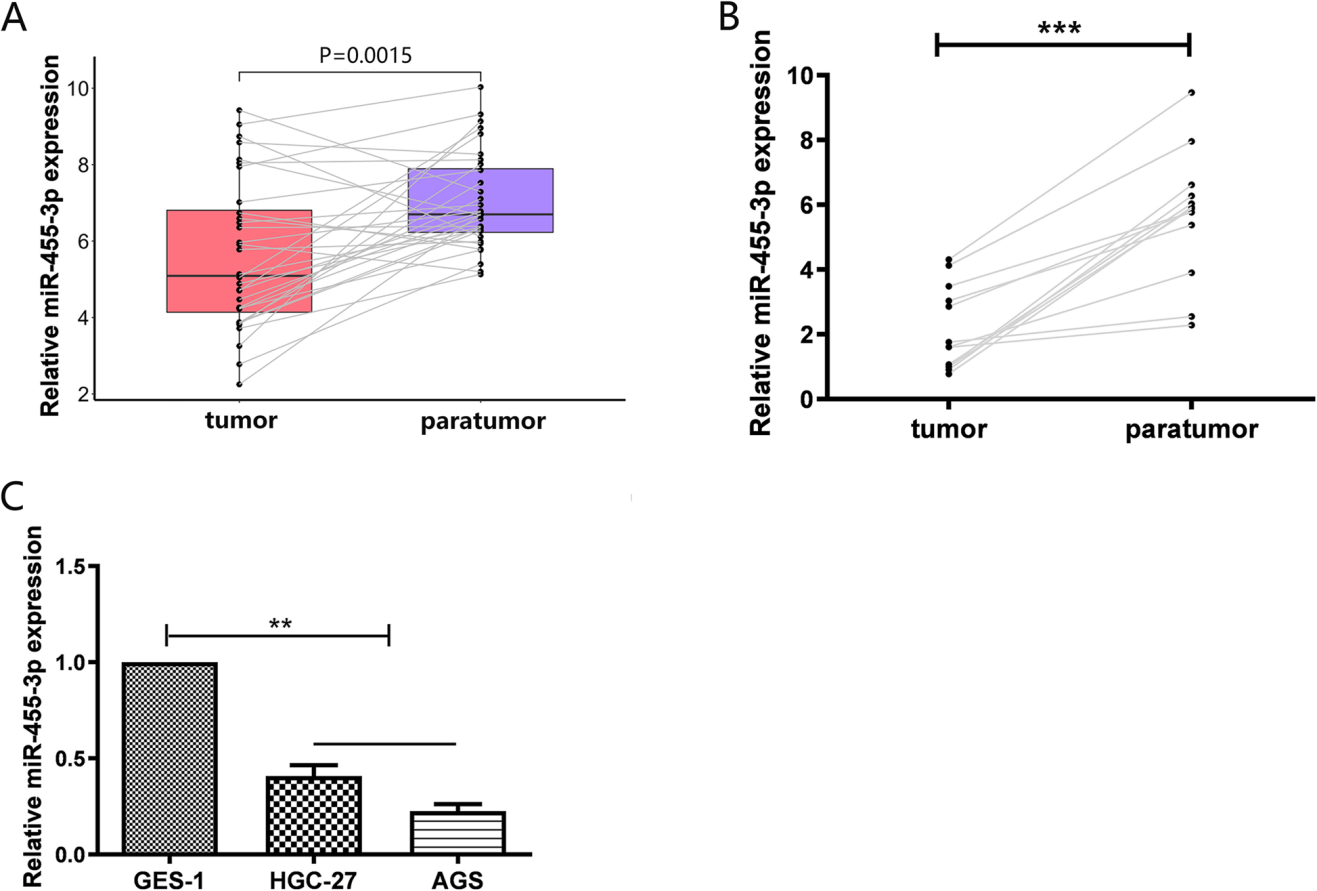
Expression of miR-455-3p is altered in GC tissues and cells (**A**) Expression level of miR-455-3p in GC tissues compared with adjacent non-tumor tissues in TCGA STAD dataset. **B** Expression of miR-455-3p in in human GC tissues and adjacent tissues. **C** Expression of miR-455-3p in GES-1, HGC-27, and AGS cells. Data are representative of three independent experiments. Statistical significance was evaluated using Student’s t tests. Data are presented as the mean ± SD. *p < 0.05, **p < 0.01, ***p < 0.001

To examine the expression of miR-455-3p in GC, 32 patient samples and paired neighboring tissue from TCGA STAD cohort were used. We observed the expression of miR-455-3p was statistically significant attenuated in tumor samples when compared with noncancerous samples (Fig. 1A). Then miR-455-3p levels in GC specimens and adjacent tissues were analyzed by qPCR. As shown (Fig. 1B), the expression of miR-455-3p in 13 GC tissues were significantly lower than those in adjacent tissues. Also, miR-455-3p levels in normal gastric epithelial cells (GES-1) were much higher when compared with AGS and HGC-27 cells (Fig. 1C). Thus, miR-455-3p may participate in GC pathophysiology.

### In GC, mir-455-3p suppresses EMT and proliferation but enhances apoptosis

**Fig. 2:**
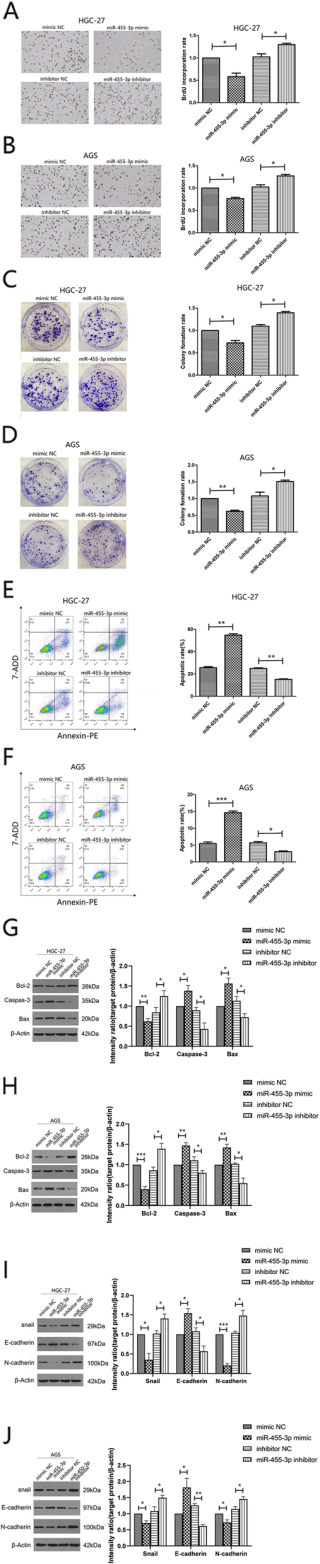
MiR-455-3p inhibits cell proliferation and EMT but promotes apoptosis in GC (**A** - **D**) The effects of miR-455-3p on proliferation in HGC-27 and AGS cells transfected with miR-455-3p mimics or inhibitors via EdU incorporation assay(Magnification 200×) (A, B) and colony formation assays (**C**, **D**). **E**, **F** of Apoptosis in HGC-27 and AGS transfected with miR-455-3p mimics or inhibitors assessed by Flow cytometry. The total events shown in the lower right-hand and upper right-hand quadrants are apoptotic cells. **G**-**J** Western blotsshowing protein levels of Bcl-2, Caspas-3, Bax, E-cadherin N-cadherin and Snail in HGC-27 and AGS cells transfected with miR-455-3p mimics or inhibitors. β-actinis a loading control. Data are representative of three independent experiments. The statistical significance was evaluated using Student’s t tests. Data are presented as the mean ± SD. *p < 0.05, **p < 0.01, ***p < 0.001

To assess functions of miR-455-3p in GC, AGS and HGC-27 cells were transfected with miR-455-3p inhibitors, mimics, and negative controls (NCs). EdU incorporation showed that DNA replication was greatly attenuated in the presence of miR-455-3p mimics, whereas miR-455-3p inhibitors increased this replication (Fig. 2A, B). Colony formation assays were also consistent with EdU incorporation assays (Fig. 2C, D). Next, we used flow cytometry to examine apoptosis; miR-455-3p overexpression increased apoptotic cell numbers in AGS and HGC-27 cells, while miR-455-3p suppression showed the opposite effects (Fig. 2E, F). To further investigate the impact of miR-455-3p on GC apoptosis, we examined gene expression (Caspase-3, Bax, and Bcl-2) associated with apoptosis mechanisms by WB. Overexpressed miR-455-3p enhanced Caspase-3 and Bax expression but diminished Bcl-2 expression, while attenuation of miR-455-3p exhibited the opposite effects (Fig. 2G, H). To determine if miR-455-3p was involved in EMT, we examined EMT-related protein expression (Snail, N-cadherin, and E-cadherin) by WB. Overexpressed miR-455-3p increased levels of E-cadherin expression levels but lowered Snail and N-cadherin. In contrast, repression of miR-455-3p lowered E-cadherin expression but enhanced Snail and N-cadherin levels (Fig. 2I, J). Thus, miR-455-3p may be implicated in GC proliferation, EMT, and apoptosis.

### In GC, ARMC8 is a direct mir-455-3p target by binding to its 3’untranslated region

**Fig. 3:**
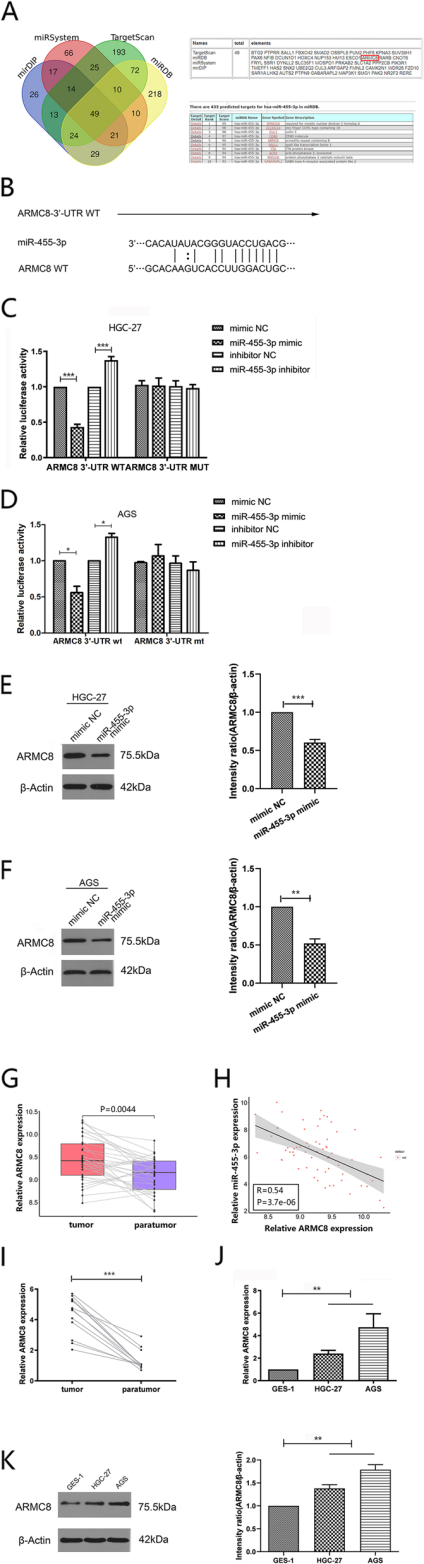
ARMC8 is a direct target of miR-455-3p by binding to 3’ UTR region in GC 9 (**A**) Online databases were used to predict target genes, followed by intersection analyses. ARMC8 have a high target scores in the miRDB database. **B **The ARMC8 3’UTR contained potential binding sites for miR-455-3p. **C**, **D** HGC-27 and AGS cells were co-transfected with miR-455-3p mimics or inhibitors and wild-type (WT) or mutant (MT) ARM8 3’UTR plasmids. Dual luciferase activity was determined after 48 h. **E**, **F** Analyses of ARMC8 expression in HGC-27 and AGS cells transfected with miR-455-3p mimic. β-actin is a loading control. **G** Expression level of ARMC8 in GC tissues compared with adjacent non-tumor tissues in The Cancer Genome Atlas dataset. **H** The relationship between ARMC8 and miR-455-3p are representative. **I** Expression of ARMC8 in human GC tissues and adjacent tissues. **J**, **K** Expression of ARMC8 in GES-1, HGC-27, and AGS cells was detected by qPCR and western blotting. Data are presented as the mean ± SD. *p < 0.05, **p < 0.01, ***p < 0.001

To further investigate downstream target genes of miR-455-3p, we used online miRSystem, miRDIP, miRDB, and TargetScan databases to predict target genes. Intersection analyses identified 49 target genes, from which, ARMC8 was selected due to its high target scores in the miRDB database (Fig. 3A).

As shown (Fig. 3B), from bioinformatics predictions, a binding site of miR-455-3p was identified in the 3’-UTR of ARMC8 mRNA. To experimentally verify this binding, relative luciferase activities were examined in HGC-27 and AGS cells, and showed that miR-455-3p markedly suppressed wild-type ARMC8 3´UTR luciferase activity but not mutant activity (Fig. 3C, D). Additionally, in AGS and HGC-27 cells transfected with miR-455-3p mimics, decreased ARMC8 protein levels were observed (Fig. 3E, F). We next analyzed ARMC8 gene expression in 32 pairs of GC tissues and paired adjacent tissue samples in TCGA STAD cohort, and showed the ARMC8 gene was overexpressed in tumor samples when compared with paired samples (Fig. 3G), and that ARMC8 expression was adversely linked to miR-455-3p expression levels in GC (r=-0.54, *P* < 0.001) (Fig. 3H). We next examined ARMC8 levels in GC cells and tissue and observed that protein and mRNA levels of ARMC8 were higher (Fig. 3I-K). Thus, miR-455-3p repressed GC by directly binding to the 3’UTR of ARMC8 mRNA, with its expression adversely linked to ARMC8 expression.

### MiR-455-3p acts as a tumor inhibitor by targeting ARMC8

**Fig. 4:**
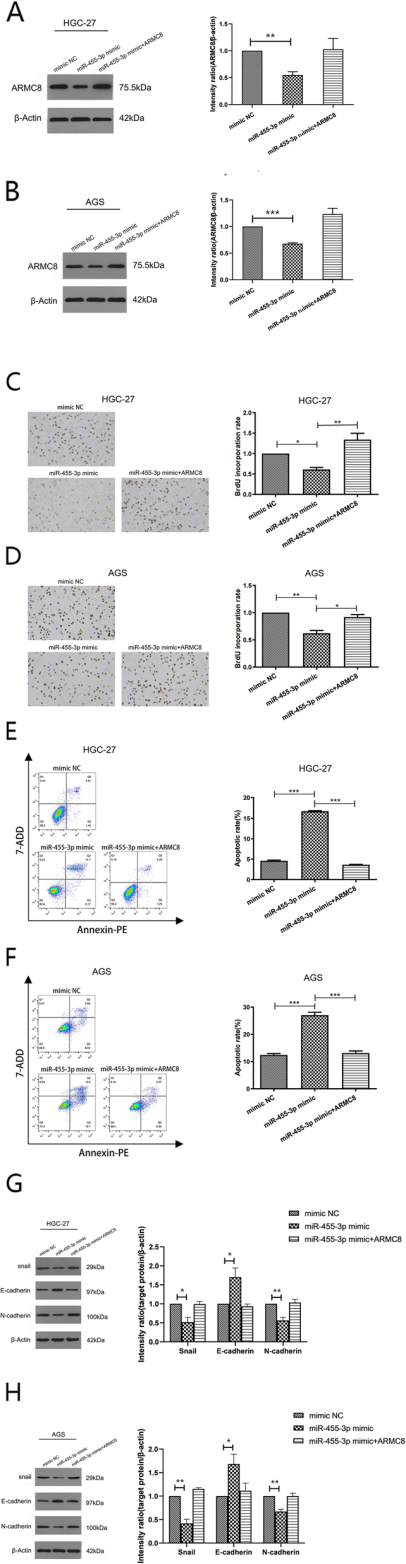
MiR-455-3p functions as a tumor suppressor via targeting ARMC8 in GC (**A**, **B**) Western blots showing ARMC8 expression in HGC-27 and AGS cells transfected with miR-455-3p mimic or miR-455-3p mimic plus ARMC8 expression vector (Magnification 200×) (**C**, **D**) EdU incorporation assay data showing cell proliferation of HGC-27 and AGS cells transfected with miR-455-3p mimic or miR-455-3p mimic plus ARMC8 expression vector. **E**, **F** Flow cytometry data showing apoptosis in HGC-27 and AGS cells transfected with miR-455-3p mimic or miR-455-3p mimic plus ARMC8. Total events shown in the lower right-hand and upper right-hand quadrants are apoptotic cells. **G**, **H** Protein levels of E-cadherin N-cadherin and Snail were determined by using western blotting. Data are presented as the mean ± SD. *p < 0.05, **p< 0.01, ***p < 0.001

To test our observations, we transformed GC cells with miR-455-3p mimics alone or miR-455-3p mimics plus an ARMC8 expression vector. From WB, ARMC8 expression was repressed by miR-455-3p but rescued by increasing ARMC8 expression (Fig. 4A, B). As anticipated, EdU incorporation was attenuated in cells transfected with miR-455-3p mimics, however, overexpressed ARMC8 antagonized the impact of miR-455-3p on cell proliferation (Fig. 4C, D). Additionally, miR-455-3p overexpression contributed to apoptosis, which was alleviated after co-transfection with an ARMC8 expression vector (Fig. 4E, F). As shown (Fig. 4G, H), overexpressed miR-455-3p enhanced protein levels of E-cadherin and inhibited levels of N-cadherin and Snail, but these changes were reversed by increased ARMC8 expression. Therefore, miR-455-3p was involved in GC proliferation, apoptosis, and EMT via binding to ARMC8.

### MiR-455-3p functions in a GC mouse model

**Fig. 5:**
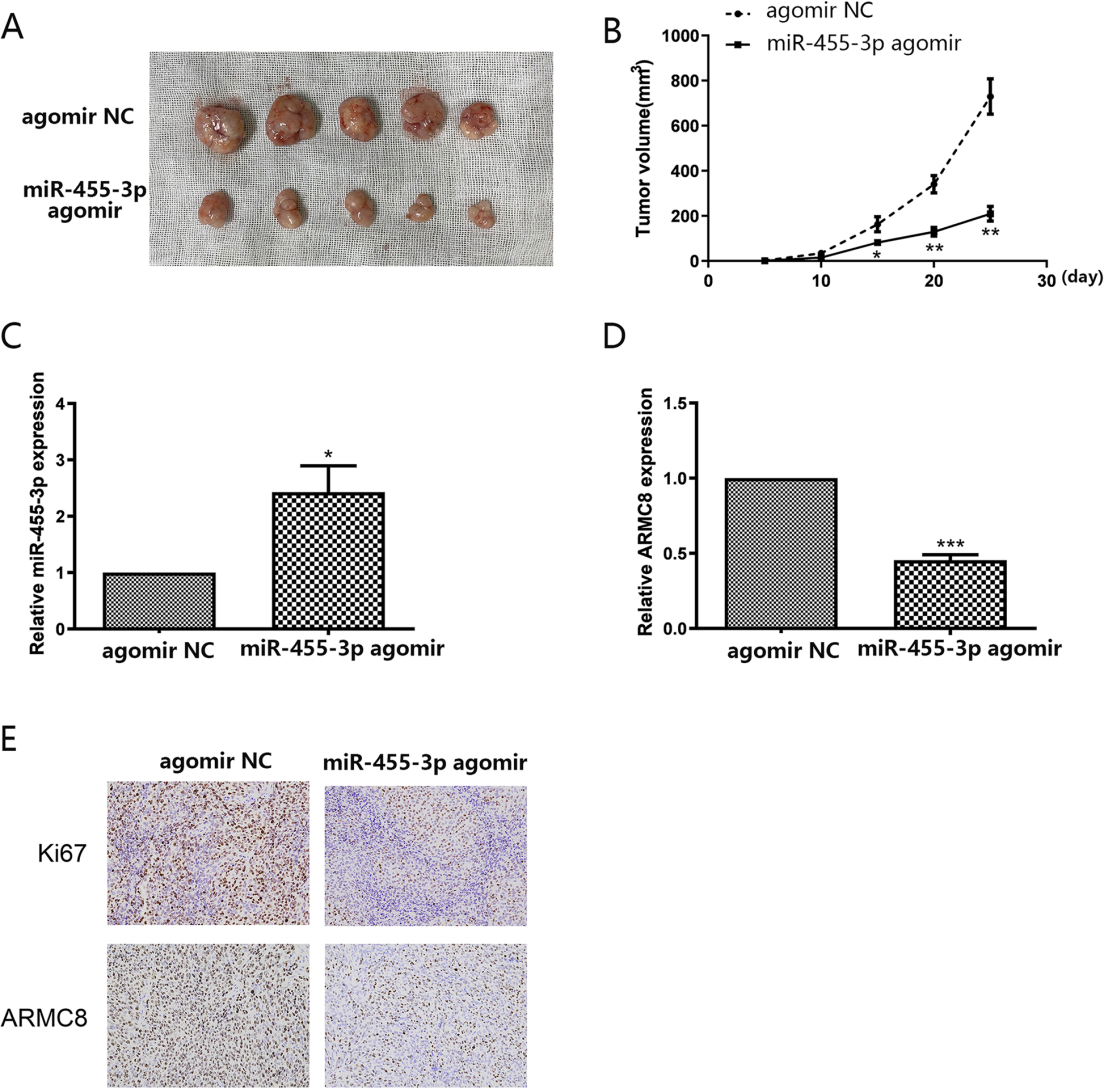
MiR-455-3p functions in vivo (**A**–**B**) miR-455-3p significantly inhibited the growth of xenografts formed by HGC-27 when compared with the NC group. **A** Representative images showing tumors isolated from tumor-bearing mice. **B** Tumor volume in nude mice. (*n* = 5 mice/group). **C** Expression of miR-455-3p was detected by qPCR. **D** Expression of ARMC8 mRNA was detected by qPCR. (E) The protein expression of Ki67 and ARMC8 was detected by IHC (Magnification 200×). Data are presented as the mean ± SD. *p < 0.05, **p < 0.01, ***p < 0.001

To investigate if miR-455-3p influenced GC progression, we subcutaneously injected HGC-27 cells into BALB/c nude mice to construct a xenotransplantation model, and subjected xenograft tumors to a miR-455-3p agomir or contrast agent. Xenograft growth was dramatically repressed by the miR-455-3 agomir in contrast to the NC agomir (Fig. 5A, B). Our qPCR analyses indicated that miR-455-3p expression was markedly elevated in tumor tissue upon miR-455-3p agomir treatment (Fig. 5C). In contrast, ARMC8 expression was downregulated (Fig. 5D). Moreover, immunohistochemistry showed lower levels of ARMC8 in xenografts upon miR-455-3p agomir treatment in contrast to the NC agomir, and also decreased levels of Ki-67 which served as a proliferation index (Fig. 5E). These findings showed miR-455-3p inhibited GC progression in vivo.

### MiR-455-3p inactivates Wnt/β-catenin signaling by binding to ARMC8

**Fig. 6:**
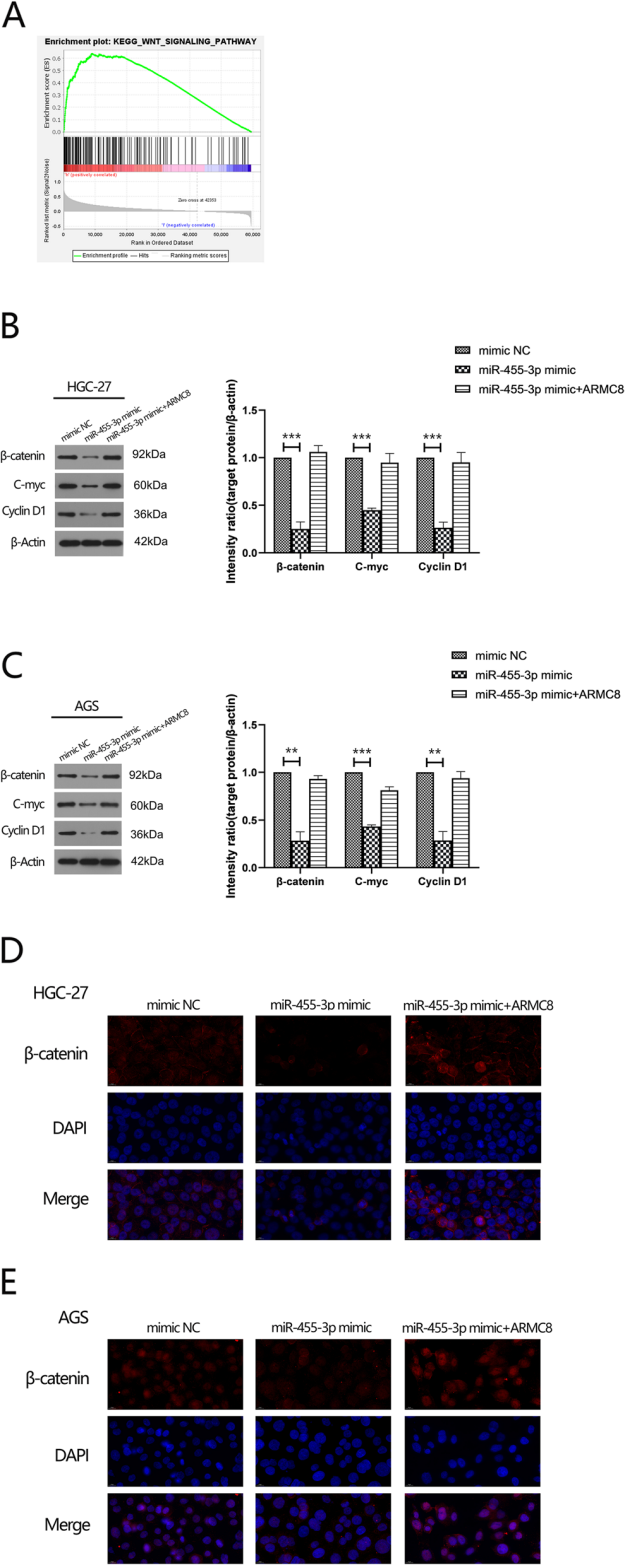
miR-455-3p represses the activation of Wnt/β-catenin pathway by binding to ARMC8 (**A**) Gene Set Enrichment Analysis evaluating ARMC8 expression and the Wnt signaling pathway in GC. **B**, **C** Protein levels of β-catenin, cyclinD1, C-myc were determined by western blotting in HGC-27 and AGS cells transfected with miR-455-3p mimic or miR-455-3p mimic plus ARMC8 expression vector. β-actin is a loading control. **D**, **E** Immunofluorescence staining results showing the distribution of β-catenin in HGC-27 and AGS cells (Scale bar: 10 μm)

To identify potential downstream pathways of ARMC8, GSEA was used to examine signaling pathways which were differentially enriched in patients with GC, with low and high ARMC8 expression, in TCGA cohort. The Wnt signaling pathway was significantly enriched for high ARMC8 expression (Fig. 6A). Therefore, we evaluated if miR-455-3p and ARMC8 could repress or activate Wnt/β-catenin signaling. Two GC cell lines were transfected with miR-455-3p mimics or miR-455-3p mimics plus an ARMC8 expression vector, and the expression of β-catenin and downstream molecules examined by WB. When compared with the NC group, protein levels of C-myc, cyclin D1, and β-catenin were decreased in the miR-455-3p mimic group, while the miR-455-3p mimic plus ARMC8 group showed no significant differences in expression (Fig. 6B, C). Also, by immunofluorescence assay, we further verified that miR-455-3p attenuated protein expression of β-catenin, while ARMC8 overexpression reversed this effect, not only elevated β-catenin protein levels but also promoted its translocation into the nucleus (Fig. 6D, E). These data indicated that Wnt/β-catenin activation was suppressed by miR 455-3p by targeting ARMC8.

## Discussion

Pathological processes in GC are extremely complex and are characterized by multiple pathogenic factors and slow progression over multiple stages [20] Aberrant molecular signaling pathways, epigenetic alterations, and gene mutations are all implicated in GC development and progression [21]. However, despite continuous improvements in medical care, GC survival rates are dismal [22], therefore identifying novel treatment targets is crucial.

Recently, aberrant miRNA expression was correlated with tumor growth and progression, including GC [23]. MiRNAs promoted or repressed GC progression by altering oncogene expression and other oncogenic factors [24, 25]. Recently, considerable attention has focused on miR-455-3p in malignant tumors; miR-455-3p expression was downregulated in colorectal cancer, while cell proliferation was inhibited by attenuating TPT1 expression [8]. Microarray analyses showed that in sera from 194 patients with breast cancer, miR-455-3p expression was considerably attenuated [9]. MiR-455-3p also restrained pancreatic cancer progression by inactivating Wnt/β-catenin signaling through TAZ [26]. Equally, miR-455-3p restrained non-small cell lung cancer cell proliferation and migration by directly binding to HOXB5 targeting and was a separate prognostic factor [27]. In osteosarcoma, miR-455-3p acted as a progressive prognostic indicator by promoting apoptosis and inhibiting malignant behaviors in cancer cells [28]. These studies exemplified how miR-455-3p may exert inhibitory roles in several malignant tumors by regulating different target genes. However, in GC, functions of miR-455-3p are poorly understood.

We confirmed that miR-455-3p was attenuated in GC. Overexpression of miR-455-3p increased apoptosis, inhibited EMT and GC cell proliferation, and suppressed tumorigenicity in vivo, while weakening the expression of miR-455-3p exhibited the opposite effects. Combined, miR-455-3p may function as a tumor suppressor, with key roles in GC progression.

To explore the molecular mechanisms by which miR-455-3p exerts these effects, we identified ARMC8 as a potential target gene of miR-455-3p. ARMC8 is a novel armadillo repeat containing protein, containing 14 armadillo (ARM) repeats, which are involved in different cellular functions, such as cell–cell adhesion, intracellular transport, signaling and ciliogenesis [16]. The human ARMC8 gene is located on chromosome 3. Notably, a chemokine receptor gene cluster and several human cancer-related loci are found on chromosome 3 [29]. Human ARMC8 has also been associated with the degradation of αE-catenin, one of the key components of the E-cadherin/β-catenin/α-catenin (CCC) complex [17]. ARMC8 was reported to contribute to malignancy in ovarian [17], hepatocellular [15], colon [16], breast cancers [29], and so on. Knockdown of ARMC8 in the hepatocellular carcinoma HepG2 cell line significantly up-regulated the expression levels of E-cadherin, β-catenin and αE-catenin [15]. Silencing of ARMC8 inhibited TGF-β-induced EMT in bladder carcinoma UMUC3 cells [30]. To further analyze molecular mechanisms, we used GSEA to show that Wnt was an underlying signaling pathway associated with ARMC8 in GC. Wnt/β-catenin signaling is involved in human cancer pathogenesis and progression [31]. The canonical Wnt pathway is also known as the Wnt/β-catenin pathway. Upon activation, the pathway triggers β-catenin stabilization and facilitates its transfer from the cytoplasm to nucleus, where it activates several genes (e.g., TCF/LEF), eventually initiating downstream gene transcripts, including matrix metalloproteinase (MMP), cyclin D1, and c-myc [32–34]. In our study, overexpressed miR-455-3p repressed the expression of c-myc, cyclin D1, and β-catenin, and also the translocation of β-catenin from the cytoplasm to the nucleus in GC cells, while overexpressed ARMC8 reversed these effects. Thus, miR-455-3p silenced the expression of ARMC8, restrained EMT and cell proliferation, and facilitated GC apoptosis by restraining Wnt/β-catenin signaling. Consequently, miR-455-3p repressed Wnt/β-catenin signaling by binding to ARMC8, thereby exerting tumor inhibition effects.

Collectively, we confirmed that miR-455-3p was low expressed in GC and ARMC8 was high expressed in GC. We suggested that miR-455-3p directly targeted ARMC8 to regulate cell proliferation, apoptosis, and EMT in GC. Furthermore, we identified a novel mechanism whereby miR-455-3p repressed Wnt/β-catenin signaling by silencing ARMC8 expression, and thus miR-455-3p suppressed GC progression. More importantly, a targeted intervention of the miR-455-3p / ARMC8/Wnt/βcatenin signaling axis could enhance GC prognosis outcomes and improve survival rates for patients with GC .

## Supplementary Information

**Additional file 1.**

## Data Availability

The mRNA-sequencing and miRNA-sequencing data of STAD were retrieved from TCGA database portal (https://portal.gdc.cancer.gov/). The data that support the findings of this study are openly available in figshare, DOI: 10.6084/m9.figshare.22298101.v1.
