# Supplementary material for: MiR-455-3p inhibits gastric cancer progression by repressing Wnt/β-catenin signaling through binding to ARMC8
Source: BMC Med Genomics. 2023 Jul 3;16:155. doi: 10.1186/s12920-023-01583-y (PMC10318695; doi:10.1186/s12920-023-01583-y)

Fig2

miRNA mimics or inhibitors were transfected into HGC-27 cells. WB was used to detect expression levels of Bcl-2, Bax, Caspase-3, E-cadherin, N-cadherin, Snail, and β-actin.


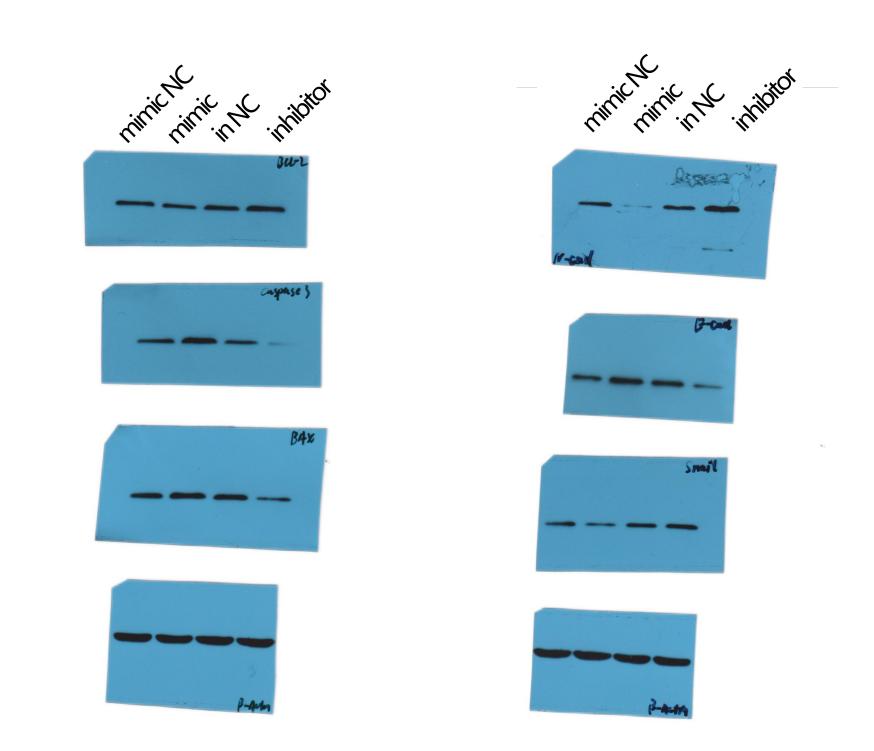


miRNA mimics or inhibitors were transfected into AGS cells. WB was used to detect expression levels of Bcl-2, Bax, Caspase-3, E-cadherin, N-cadherin, Snail, and β-actin.


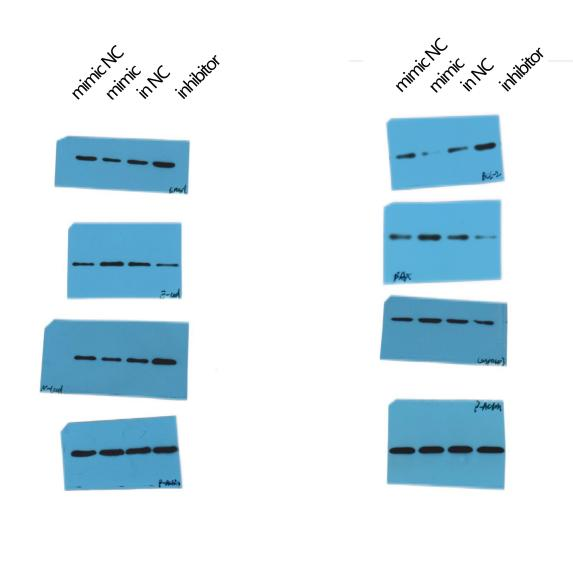


Fig 3

miRNA mimics and NC were transfected into HGC-27 cells. WB was used to detect expression levels of ARMC8. (On the left is HGC-27，the right is AGS）


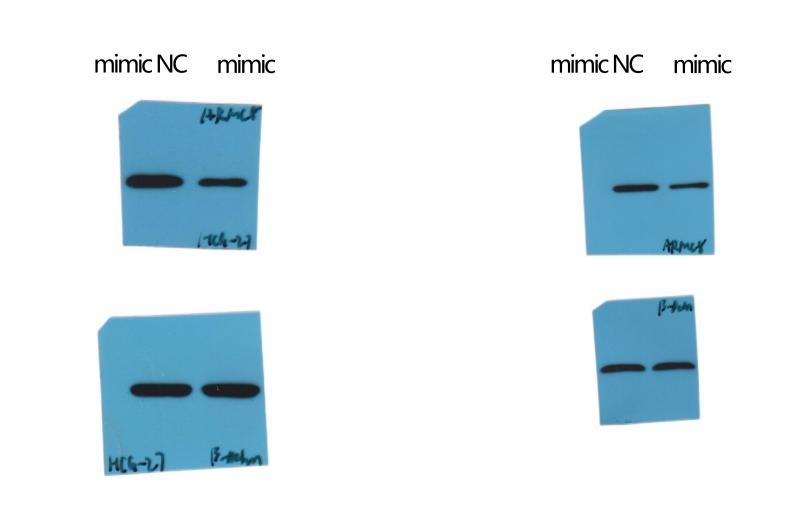


Expression levels of ARMC8 by WB in three cells: GES-1, HGC-27, and AGS


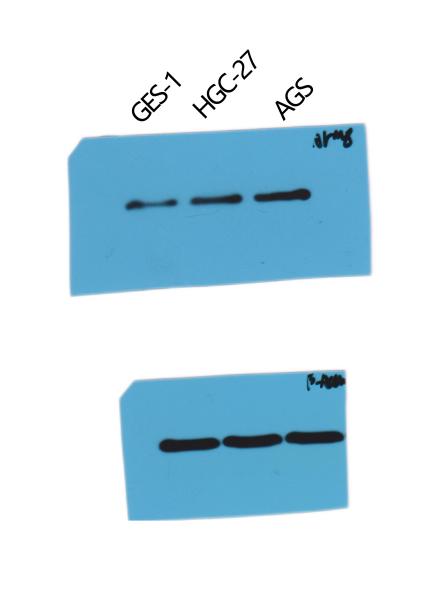


Fig 4

After transfected GC cells with miR-455-3p mimics or miR-455-3p mimics plus the ARMC8 expression vector, WB was used to detect expression levels of ARMC8. (On the left is HGC-27, the right is AGS）


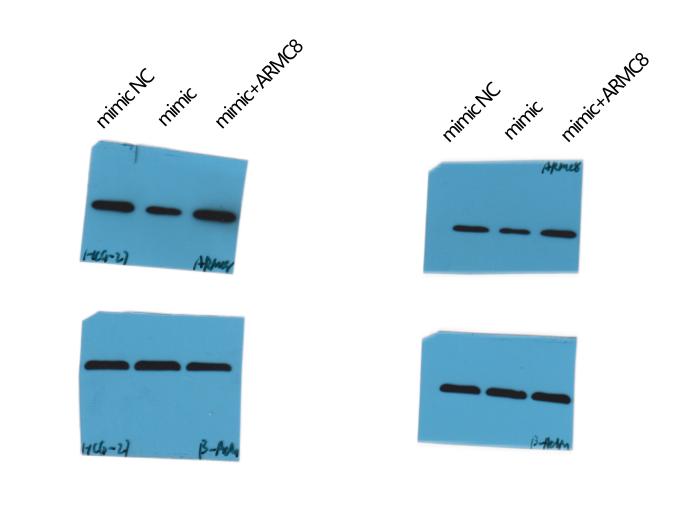


After transfected GC cells with miR-455-3p mimics or miR-455-3p mimics plus the ARMC8 expression vector, WB was used to detect expression levels of E-cadherin, N-cadherin, Snail, and β-actin. (On the left is HGC-27, the right is AGS）


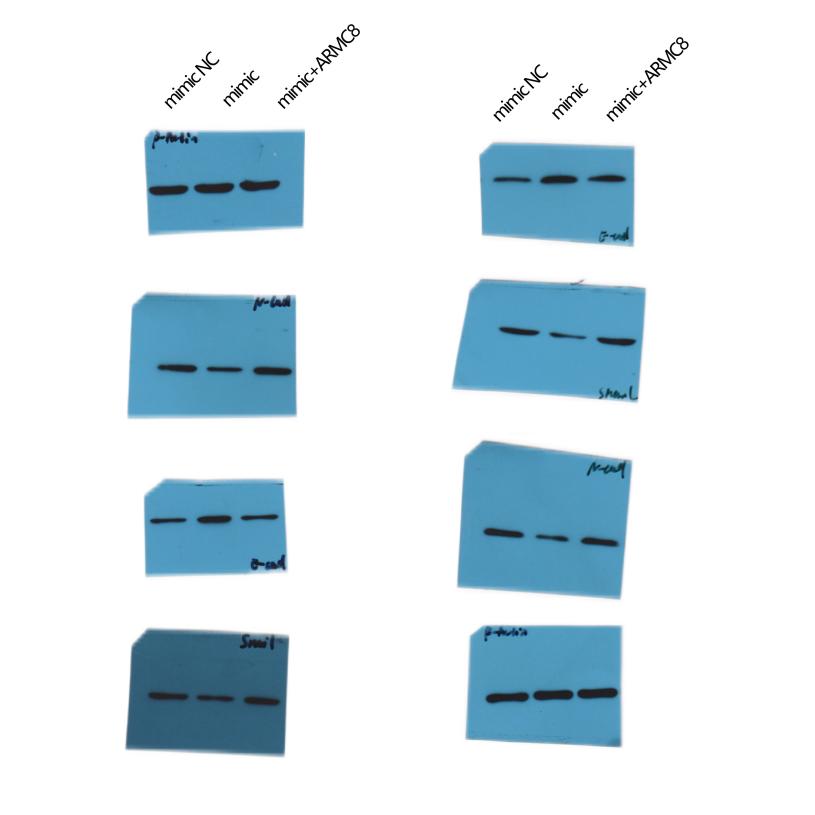


Fig 6

After transfected GC cells with miR-455-3p mimics or miR-455-3p mimics plus the ARMC8 expression vector, WB was used to detect expression levels of β-catenin, cyclinD1, C-myc, and β-actin. (On the left is HGC-27, the right is AGS）


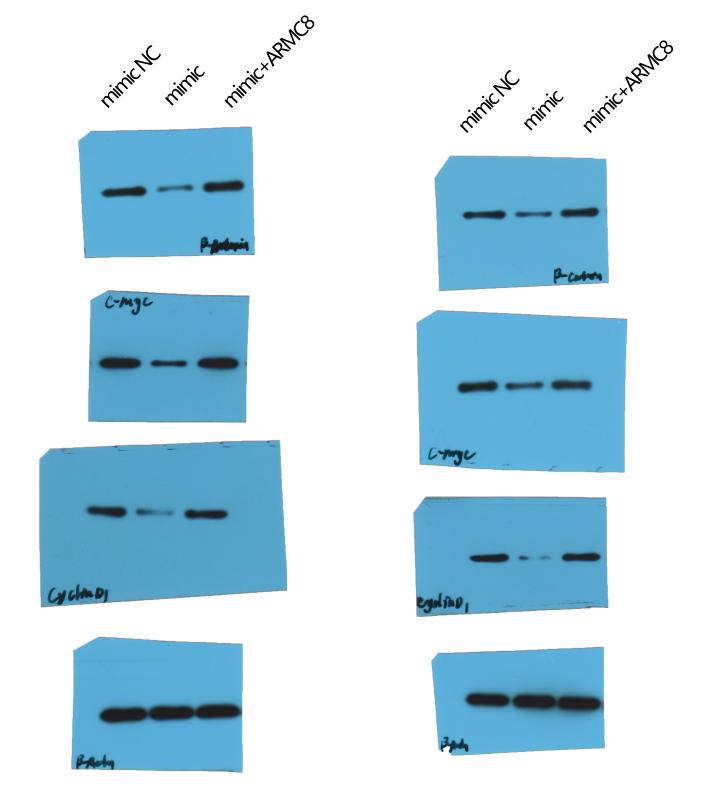

Supplement: Supplementary file 1 — Additional file 1. [file 12920_2023_1583_MOESM1_ESM.docx]
